# Role of Vitamin A/Retinoic Acid in Regulation of Embryonic and Adult Hematopoiesis

**DOI:** 10.3390/nu9020159

**Published:** 2017-02-20

**Authors:** Ana Cañete, Elena Cano, Ramón Muñoz-Chápuli, Rita Carmona

**Affiliations:** 1 Department of Animal Biology, Faculty of Science, University of Malaga, Campus de Teatinos s/n Malaga 29071, Spain and Andalusian Center for Nanomedicine and Biotechnology (BIONAND), Severo Ochoa 25, Campanillas 29590, Spain; acansan@uma.es (A.C.); chapuli@uma.es (R.M.-C.); 2 Max-Delbruck Center for Molecular Medicine, Robert Roessle-Strasse 10, 13125 Berlin, Germany; Elena.CanoRincon@mdc-berlin.de

**Keywords:** vitamin A, retinoic acid, hematopoiesis, embryos, leukemia, vitamin A deficiency

## Abstract

Vitamin A is an essential micronutrient throughout life. Its physiologically active metabolite retinoic acid (RA), acting through nuclear retinoic acid receptors (RARs), is a potent regulator of patterning during embryonic development, as well as being necessary for adult tissue homeostasis. Vitamin A deficiency during pregnancy increases risk of maternal night blindness and anemia and may be a cause of congenital malformations. Childhood Vitamin A deficiency can cause xerophthalmia, lower resistance to infection and increased risk of mortality. RA signaling appears to be essential for expression of genes involved in developmental hematopoiesis, regulating the endothelial/blood cells balance in the yolk sac, promoting the hemogenic program in the aorta-gonad-mesonephros area and stimulating eryrthropoiesis in fetal liver by activating the expression of erythropoietin. In adults, RA signaling regulates differentiation of granulocytes and enhances erythropoiesis. Vitamin A may facilitate iron absorption and metabolism to prevent anemia and plays a key role in mucosal immune responses, modulating the function of regulatory T cells. Furthermore, defective RA/RARα signaling is involved in the pathogenesis of acute promyelocytic leukemia due to a failure in differentiation of promyelocytes. This review focuses on the different roles played by vitamin A/RA signaling in physiological and pathological mouse hematopoiesis duddurring both, embryonic and adult life, and the consequences of vitamin A deficiency for the blood system.

## 1. Introduction

Vitamin A was discovered in the second decade of the twentieth century by Elmer McCollum and Marguerite Davis [1]; also known as retinol, it is one of the fat soluble vitamins, and plays an important role in vision, reproduction, immune function, as well as cell growth and communication [2,3,4,5,6,7,8,9,10]. Vitamin A, regarded as an important micronutrient in mammalian diet, exists in three forms: retinal, retinol and retinoic acid (RA), the latter being the most metabolically active. Dietary vitamin A is obtained from plant sources (provitamin A carotenoids, particularly β-carotene) or as retinyl esters from animal sources, fortified food products and supplements. The metabolic fate of this retinol is the esterification and tissue storage (primarily in the liver), or the irreversible oxidation to all*-trans* retinaldehyde and to all-*trans* retinoic acid (ATRA) by alcohol and aldehyde deshydrogenases. In mammals, this latter family of enzymes includes three cytosolic aldehyde dehydrogenases: RALDH1 (encoded by the *ALDH1A1* gene) [11], RALDH2 (*ALDH1A2*), which is the main RA-synthesizing enzyme in the mesoderm [12], and RALDH3 (*ALDH1A3*) [13,14,15].

**Figure 1:**
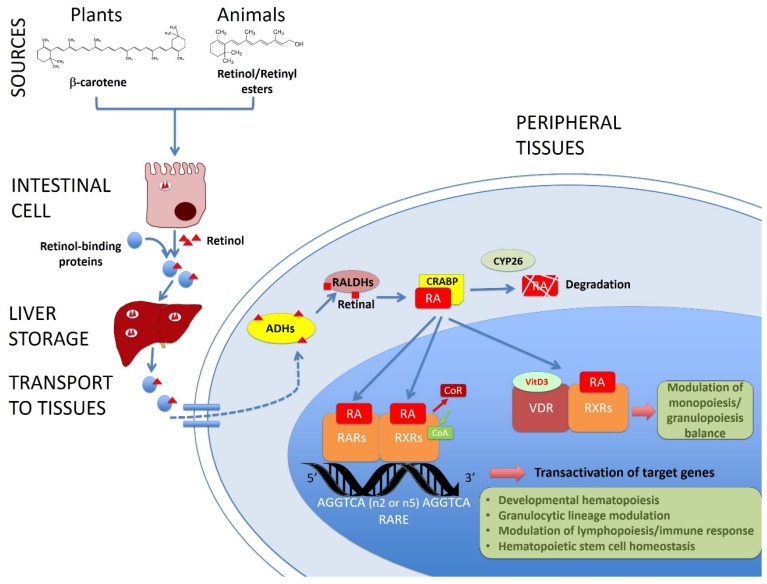
Role of vitamin A/retinol in adult hematopoiesis. This picture shows the main molecular pathways leading from the vitamin A sources to the target genes of the retinoic acid (RA, the active form of vitamin A) related with hematopoiesis in the tissues. Retinol or provitamin A is ingested and absorbed through the intestine, transported by retinol-binding proteins and stored in the liver. Retinol is transformed by the cells into RA by alcohol and aldehyde dehydrogenases (ADHs and RALDHs respectively). RA is transported by cellular retinoic acid binding proteins (CRABP) and it can be degraded by CYP26 or translocated to the nucleus, where it binds and activates nuclear retinoid acid receptors (RARs and RXRs), displacing co-repressors and recruiting coactivators of the transcription of target genes. In this way, RA regulates the developmental hematopoiesis, modulates lympho and granulopoiesis and contributes to the homeostasis of the hematopoietic stem cells. Vitamin D receptor (VDR) can also dimerize with RXRs and modulate the immune response.

RA signaling is mediated by two families of nuclear receptors: RARs and RXRs, including three members α, β and γ for each family [16]. Nuclear retinoid receptors are frequently composed of RXR and RAR heterodimers, although RXRs can form homodimers or heterodimers with other nuclear receptors such as the vitamin D receptor, as discussed below, or PPARs (peroxisome proliferator-activated receptors). In the nucleus, the RAR/RXR complex is bound to a specific sequence of DNA (RARE: retinoic acid response element) and usually performs repressor roles in the absence of ligands, activating transcription of target genes when bound to them ([16,17]) (Figure 1).

Vitamin A deficiency is widespread in developing countries [8]. Besides prominent ocular consequences of this deficiency (e.g., xerophthalmia), anemia and immune deficiency highlight other critical roles of vitamin A/RA signaling [5,6]. Furthermore, RA signaling is crucially involved in the pathogenesis but also in the treatment of some types of leukemia [18,19]. For these reasons, in this review we aim to provide the readers with an update on the functions played by vitamin A/RA signaling in the development, homeostasis and pathology of the hematopoietic system. An earlier report of our group mainly focused on the functions of RA signaling for developmental hematopoiesis [20]. In this review, mainly focused on the mouse model, we pay more attention to essential roles played by vitamin A in the hematopoietic system. 

## 2. Vitamin A/Retinoic Acid in Developmental Hematopoiesis

**Figure 2:**
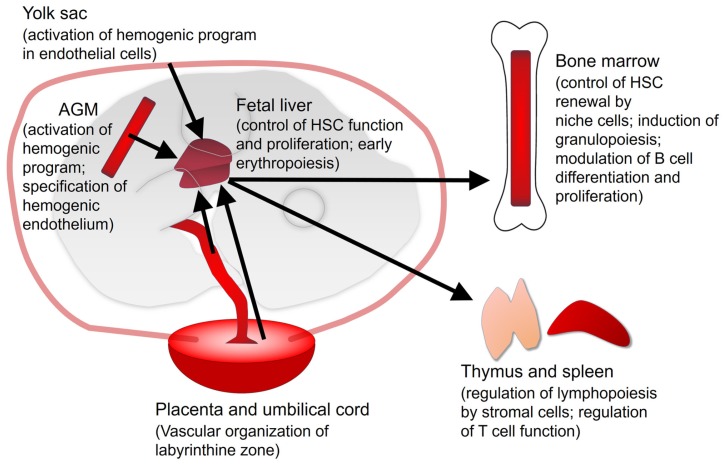
Hematopoietic sites in the embryo (**left**) and adult mouse (**right**). The main functions played by vitamin A/RA signaling are described between parentheses. Black arrows represent the main routes of migration of progenitors between the hematopoietic organs and tissues. Exchange of progenitors between embryonic organs is also possible. It is unknown if RA signaling plays an intrinsic role on placental hematopoietic stem cells (HSC). AGM: Aorta-Gonad-Mesonephros.

RA signaling plays an essential role in vascular development and in the early embryonic hematopoiesis. In the mouse embryo, hematopoiesis starts around E7.0–E7.5, when a first generation of primitive erythroid cells and endothelial cells forms clusters or blood-islands in the splanchnic mesoderm of the yolk sac. Later, these clusters connect with the developing vessels of the embryo. These primitive erythroid cells are soon replaced by a second wave of hematopoiesis, when hematopoietic progenitors and the definitive hematopoietic stem cells (HSC) arise from hemogenic endothelial cells [21,22,23]. This second wave gives rise to different blood cell lineages and originates sequentially in precise mesodermal domains of the mouse embryo, the yolk sac first, by E8.5, the placenta by E9.5 and finally in the aorta-gonad-mesonephros (AGM) area by E10.5 [24,25]. Later, about E11–E12, the fetal liver becomes the main site for embryonic hematopoiesis, receiving progenitors from the mesodermal domains quoted above and allowing for their expansion. The HSC and the hematopoietic activity finally move to the bone marrow shortly before birth [26,27]. As follows, we will describe how signaling by RA acts in the main hematopoietic sites: yolk sac, AGM, fetal liver and placenta (Figure 2).

### 2.1. Yolk Sac

RA signaling is involved in the differentiation of the endothelium, as well as in the emergence of the hematopoietic progenitors [28]. The embryonic endothelium of the yolk sac expresses RARα, and this expression is prominent in FLK1+/c-KIT+/CD45− cells that represent the hemogenic endothelium [29]. 

RALDH2 is expressed in visceral endoderm and generates RA, which activates the RARs of the visceral mesoderm. The lack of RA in RALDH2 knockout mice leads to death at E10.5 due to multiple defects, including disorganization of yolk sac extraembryonic vascular network [29,30,31,32] and downregulation of hematopoiesis-related genes (such as GATA1/2, SCL/TAL1, LMO2 and RUNX1) by the hemogenic endothelium [29]. These developmental defects in the RALDH2 mutants can be rescued with exogenous RA treatment in vivo and in embryo culture [29,32].

### 2.2. Aorta-Gonad-Mesonephros (AGM)

**Figure 3:**
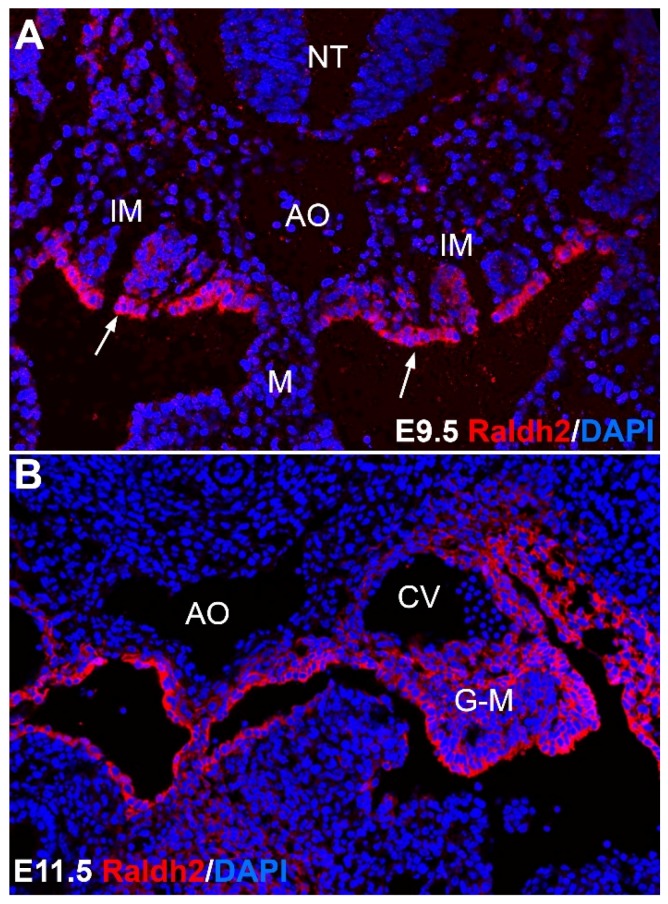
Vitamin A in developmental hematopoiesis. The picture shows immunolocalization of the enzyme RALDH2, catalyzing a key step in the generation of retinoic acid from retinol in the aorta-gonad-mesonephros. RALDH2 is expressed in the coelomic epithelium of the intermediate mesoderm (IM) (arrows in A) by the stage E9.5 (**A**) and later (**B**) in mesenchymal cells of area, where the definitive population of hematopoietic stem cells is generated, between the aorta (AO), the cardinal veins (CV) and the gonadal/mesonephric mesoderm (G-M). This embryonic RA signaling is essential for the emergence of the definitive blood progenitors, as described in the text. M: mesentery; NT: neural tube.

As well as in the yolk sac, RA signaling in AGM is essential for emergence of hematopoietic progenitors, including definitive HSC, from the hemogenic endothelium of the dorsal aorta. RALDH2 is expressed in coelomic mesothelium at E9.5 and later in mesenchymal cells of AGM region (Figure 3). Activation of RA signaling pathway enhances the generation of HSCs in both isolated hemogenic endothelial cells and pre-HSCs. In addition, conditional deletion of RALDH2 in endothelium (using a VE-Cadherin^Cre^/RALDH2^flox^ system) precludes the HSC formation in yolk sac and dorsal aorta, demonstrating that the endothelium itself is a main source of RA. These authors also demonstrated that the RA signal for the endothelial-hematopoietic transition is transduced through RARα, a receptor highly expressed in the endothelium [33]. However, the RARα knockout mice do not show an abnormal phenotype, likely due to a compensation of its function by other RA receptors [34,35].

RA signaling is also involved in the regulation of endothelial cell cycle and the specification of the hemogenic endothelium [28]. The c-KIT expression by the hemogenic endothelium (cell surface marker of hematopoietic precursors) is downregulated in RALDH2 deficient mouse embryos while RUNX1, a hematopoietic stem cell transcription factor, is upregulated. This leads to endothelial hyperproliferation and an impaired hemogenic endothelium, a phenotype very similar to the one resulting of NOTCH signaling inhibition. It has been proposed that the RA/c-KIT/NOTCH axis regulates the endothelial cell cycle and the specification of the hemogenic endothelium whereas RUNX1 regulates the generation and/or propagation of multilineage HSC from the hemogenic endothelial cells [36].

### 2.3. Fetal Liver

Fetal liver HSCs probably require RA signaling for induction of HOXA medial gene expression and control of HSC identity and function, as suggested by experiments with human embryonic stem cells and fetal liver HSC [37]. Incidentally, HOXA cluster genes, which are targets of RA signaling, regulate proliferation of adult mouse HSC [38].

RA signaling has a role in the erythropoiesis from the fetal liver. Erythropoietin (EPO) is a glycoprotein hormone produced by the kidney and promotes the formation of red blood cells by the bone marrow. The early wave of erythropoiesis in the yolk sac is EPO independent; however, the early phase of fetal liver erythropoiesis requires EPO, retinoic acid signaling through RAR/RXR receptors and also stabilization of the hypoxia inducible factor (HIF1). Interestingly, RXRα requirement is only active during this early phase (E9.5–E11.5), as erythropoiesis can continue without RXRα signaling from E12 on. In fact, EPO expression is 10 folds lower in RXRα^-/-^ fetal liver than in controls at E10, but there are no differences at E12. Other elements of the retinoic signaling pathway, such as RALDH1/2, cellular retinol-binding protein-1 (CRBP1) and cellular retinoic acid-binding protein-1 and 2 (CRABP1/2) are highly expressed in the early fetal liver, when the hematopoiesis is taking place, and are downregulated by midgestation, suggesting that they may play a role in hematopoiesis [39]. 

### 2.4. Placenta and Umbilical Cord

Hematopoiesis in the placental labyrinthine zone and umbilical arteries has been well described throughout development [22,40,41,42,43,44,45,46]. The retinoic acid receptor RXRα is strongly expressed in the developing labyrinthine zone of the chorioallantoic placenta. Inactivation of this receptor causes disorganization of the labyrinthine zone, abnormal stasis of maternal blood and a disruption of the network of embryonic vessels and maternal sinuses [47]. RXRα^-/-^/RXRβ^-/-^ double mutant embryos die between E9.5 and E10.5 due to a lack of formation of the labyrinthine zone [48]. However, a direct requirement of RA signaling on placental hematopoiesis has not been described, but it cannot be discarded. A population of hemogenic precursors expressing endothelial and early hematopoietic markers is localized in the vascular labyrinth where RA/RXR signaling is critical [49]. Thus, more studies will be necessary to know if RA plays a role in placental/umbilical cord hematopoiesis as it happens in other embryonic territories. 

### 2.5. Vitamin A/Retinoic Acid and Developmental Hematopoiesis in Zebrafish

The zebrafish (*Danio rerio*) is a powerful animal model to study the role of RA during development. Some recent reports have shown that RA signaling is critical in early steps of zebrafish hematopoiesis, including the formation of the HSCs. RA signaling is required for HSC marker expression even before the formation of the aorta, when the main RA synthesis enzyme, RALDH2, is expressed in the paraxial mesoderm. This requirement is related with the expression of junctional adhesion molecules JAM1a and JAM2a (which are essential for proper transduction of Notch signals), the chemokine CXCL12 and its main receptor CXCR4 [50]. 

Later, RA signaling is involved in the generation of a first wave of erythrocytes and myeloid cells, but playing in this case an inhibitory role. This primitive wave of hematopoiesis is strongly reduced by treatment with exogenous RA at the 5 somite stage, while expression of the vascular marker FLI1 is increased. This effect can be rescued by injection of mRNA of the hematopoietic transcription factor SCL. Inhibition of the enzyme RALDH2 by 4*N*-diethylaminobenzaldehyde (DEAB) treatment leads to increased expression of GATA1-expressing erythroid cells [51,52]. Primitive myelopoiesis is also restricted by RA before 11 hpf (hours post fertilization). Interestingly, GATA4/5/6-depleted zebrafish embryos show deficient primitive myelopoiesis but this phenotype can be rescued by DEAB treatment or RALDH2 knockdown. Thus, GATA4/5/6 are negative regulators of RA signaling, while RA inhibits commitment of mesodermal cells to hematopoietic fates upstream SCL [53].

## 3. Vitamin A/Retinoic Acid in Adult Hematopoiesis

Vitamin A (Retinol) and its derivatives (retinoids) play a key role in development and are important in the homeostasis of many organs in adults [16]. 

The receptors RARα and γ, but not the RARβ receptor, are highly expressed in the adult hematopoietic system. RARα is expressed in a variety of bone marrow cells, while RARγ is expressed in primitive hematopoietic progenitors, a deficiency of which causes a reduction in the number of HSC [54]. Loss of function of RARα and γ results in viable mice with reduced lifespan [34,35].

RARα^-/-^ mice show no defects in the early steps of adult hematopoiesis and this receptor is not involved in the self-renewal potential of HSC. However, RA signaling transduced through RARα is critically required for granulocytic lineage modulation. The presence of free RARα receptor not bound to RA inhibits neutrophil differentiation while the binding of RA induces its differentiation [55]. However, overexpression of RARα increases the number of granulocytes while overexpression of RARγ induces increase of undifferentiated progenitors [54].

RARγ^-/-^ mice are anemic [56] and develop myeloproliferative syndrome, likely by a defective function of RARγ in the bone marrow cellular niche [57]. The erythropoiesis is normal after conditional deletion of RARα and RARγ in adult cells expressing the EPO receptor (EPOR), indicating that RARγ is playing a function in cells other than erythroid progenitors [56]. NOTCH and HOXB4 are known regulators of HSC self-renewal [58,59]; while HOXB4 expression in HSC is normal in the RARγ mutants, NOTCH1 and its effector HES1 are both downregulated [54]. 

In contrast, it has been demonstrated that RARγ regulates B and T lymphopoiesis in the thymus and bone marrow. It was initially described that RARγ mutants only display defective primary and memory CD8+ T cell response, and the production of inflammatory cytokines by macrophages appears impaired [60]. However, the conditional deletion of this receptor in nestin-expressing stromal cells leads to a decrease in the number of circulating B and CD4+ T cells demonstrating that RARγ receptor expression in the bone marrow and thymus microenvironment is required for lymphopoiesis [61].

In humans, the RA/RAR signaling system also plays a key role in the balance between self-renewal and differentiation in the bone marrow niche. Bone marrow human primitive CD34+/CD38− HSC express RALDH1 and RARα. The inhibition of the RA signaling on cultured HSC induces HOXB4 upregulation, which prevents their differentiation and enhances their proliferation and expansion in an undifferentiated stage [62]. RA degradation by the enzyme CYP26 from stroma could be involved in this mechanism, contributing to the maintenance of the pool of self-renewing HSCs. On the other hand, pharmacological inhibition of aldehyde dehydrogenases with DEAB allows for expansion of HSC in culture, preventing their differentiation. Treatment with retinoids and vitamin D abolishes this effect [63]. However, when human HSC are cultured with RARα antagonists, the lifespan of the cultures, the number of the progenitors and also the production of monocytes and neutrophils increase, while antagonists of RARγ do not affect the cultures [64]. This specific role of RARα for human HSC would be different to that above described in mice.

RA/RARα signaling is also essential for lymphocyte function and plays a key role in the regulation of the immune homeostasis, the differentiation of T cell subsets, the migration of T cells into tissues, and the development of T cell-dependent antibody responses [65,66,67]. In the intestine, RA is produced by gut-associated dendritic cells and is required for generating gut-associated lymphocytes [68]. Migration of innate lymphoid cells to the intestine is induced by changes in the homing receptors regulated by mucosal dendritic cells and RA signaling [69,70]. These effects of vitamin A/RA signaling on T cell biology account for the central role played by this micronutrient in immunity [71].

RA also modulates proliferation and differentiation of B cells [72,73]. RA signaling, together with Toll-like receptor 9 (TLR9) ligands, protect B lymphocytes against damage-induced apoptosis through the increase of the expression of the myeloid cell leukemia-1 protein. Importantly, this protective effect was not observed in malignant B cells, thus suggesting that RA+TLR9 ligands could be useful in treatment of B-cell malignancies by selectively protecting non-malignant B cells [74].

## 4. RA and Leukemia

The role played by RARα in modulation of the granulocyte differentiation above described is related with the pathogeny of acute promyelocytic leukemia (APL). This disease is produced by a chromosomal rearrangement, where the RARα gene fuses with other genes and generate a dominant defective form of RARα. Lack of RA signaling through the RARα receptor results in blockage of promyelocytic progenitors’ differentiation. The most frequent translocation is the fusing of the PML gene, which codes for the promyelocytic leukemia protein, a tumor suppressor transcription factor, with the RARα gene, although up to eight genes more have been identified in these translocations [18].

The treatment of APL with all trans-retinoic acid (ATRA) combined with arsenic trioxide (As_2_O_3_, ATO) has improved dramatically the prognostic of this disease, lethal some decades ago, and currently cured in about 95% of the cases [19,75,76,77,78]. The National Comprehensive Cancer Network has adopted ATRA and ATO as first-line treatments for APL in its 2014 guidelines [79]. For non-high-risk APL patients treatment with RA+ATO without chemotherapy resulted in 2-year overall survival rates of 99% [80]. A recent trial showed 7-year survival rates above 90% when arsenic and ATRA were used as first-line target treatments (with chemotherapy for consolidation) [81]. Other clinical trials in non-high-risk patients are also promising [82,83]. For a review on ATRA/ATO treatment in high-risk patients see Norsworthy and Alman [84].

Acute myeloid leukemia (AML) is a disease characterized by accumulation of myeloid blasts in the bone marrow, blood and other tissues, which lack the ability to differentiate, consequently causing a decreased production of normal blood cells [85]. A number of synthetic or natural products are being assayed in preclinical studies to further improve the beneficial effect of the ATRA/ATO treatments [78,86,87,88]. The effect of HER2/ERBB2 inhibitor, TAK165, can be emphasized, which synergizes with ATRA, thus leading to improved differentiation of AML cells, which are normally resistant to RA treatment. HER2 is the epidermal growth factor receptor 2, which plays a critical role in the regulation of mammalian cell survival, proliferation and differentiation [89].

Despite these excellent therapeutic outcomes, treatment of leukemia with ATRA can produce inflammatory reactions in about 25% of patients. These adverse reactions are known as differentiation syndrome, previously known as ATRA syndrome, and it is characterized by fever, peripheral edema, pleuropericardial effusion, respiratory distress, hypotension and renal/hepatic dysfunction [90]. The differentiation syndrome is caused by release of inflammatory cytokines and it requires a preventive strategy based in corticosteroid treatment [91].

The molecular pathways leading to differentiation of APL cells induced by ATRA are being unveiled by studies in vitro. RA induces a neutrophil-like phenotype in HL-60 leukemia cells [92] and increases their differentiation [93]. Differentiation of NB4 cells, a maturation inducible cell line isolated from human acute promyelocytic leukemia, requires RAF1/MEK/ERK signaling involved in ATRA-induced differentiation in APL cells through enhancing the protein level of C/EBPβ, C/EBPε and PU.1 [94]. Additionally, ATRA inhibits HOXA7 in NB4 cells [95]. HOXA5 is upregulated by ATRA in K562 human myeloid leukemia cell line, provoking apoptosis and inhibition of cell cycle [96]. However, function of HOX cluster genes in leukemia cells is very complex, as demonstrated by the decreased proliferation and increased apoptosis reported in pro-monocyte U937 cells, when HOXA5 is downregulated by shRNA [97].

Other applications of RA have been reported in immunotherapy of T cell leukemia. ATRA, combined with interferon-α, upregulate CD38 in malignant cells, rendering them sensitive to a chimeric anti-CD38 antibody [98]. 

## 5. Epigenetic Modulation of Hematopoiesis by Vitamin A/RA

As described above, vitamin A/RA signaling exerts their regulation on hematopoietic processes through the transcriptional activity of the RAR and RXR receptors. However, epigenetic mechanisms also regulate hematopoiesis (reviewed in Shashida and Iwama, [99]) and are also involved in hematopoietic malignancies. Chimeric oncoproteins generated by chromosomal translocations, as described above, can induce chromatin alterations on target genes by aberrant deployment of enzymes, such as DNA methyltransferases and histone acetyltransferases, deacetylases and methyltransferases [100,101]. For example, an epigenetic modification mediates ATRA resistance in AML cells. The most common acute myeloid leukemia-associated fusion protein, AML1/ETO, forms a complex with RARα and promotes a repressed chromatin conformation at the RARβ2 regulatory regions. In fact, the treatment with the DNA methylation inhibitor 5-azacytidine, reverts this alteration and restores the differentiation response induced by ATRA treatment in the same way as the knockdown of the AML1/ETO expression [102]. Thus, lysine-specific demethylase-1 (LSD1), an enzyme responsible for demethylation of histone H3, represents a promising pharmacologic approach for AML therapy-enhancing response to ATRA treatment, and as a consequence, clinical trials are being currently performed in this direction [103]. 

One percent of APL cases are due to a translocation fusing the promyelocytic leukemia zinc finger (PLZF) gene with the RARα gene. These APL are refractory to ATRA treatment. PLZF represses the *CRABPI* locus (encoding cellular retinoic acid binding protein I) by an epigenetic mechanism involving chromatin condensation [104]. As well, PLZF-RARα interacts with histone deacetylase corepressors promoting epigenetic silencing of the cell cycle regulator p21/CDKN1A, resulting in increased proliferation [105,106]. Incidentally, p21 expression is activated by retinoic acid through a RARE in the *CDKN1A* promoter [106].

Myelofibrosis with myeloid metaplasia (MMM) is another hematopoietic malignancy related with RA signaling and its epigenetic regulation. Expression of RARβ2 is downregulated in CD34+ cells from MMM patients, but only in 25% allelic loss has occurred due to a 3p24 chromosomal deletion. Instead, hypermethylation of RARβ2 locus was found in most MMM patients but not in normal individuals [107]. This RA receptor was mentioned above as mediator of the ATRA resistance in AML1/ETO myeloid leukemia, where it is also repressed by epigenetic mechanisms.

Finally, miRNAs post-transcriptional regulation in hematopoietic cells can also play a role in retinoid-based treatment of leukemia. For example, regulation by miR-223 is essential for ATRA-induced differentiation of acute promyelocytic leukemia (APL) blasts to granulocytes [108].

## 6. Vitamin A/Vitamin D Interrelationships in Hematopoiesis and Leukemia

As stated in the introduction, RXRs can form heterodimers with the vitamin D receptor (VDR). In this way, physiological functions mediated by vitamin D, via the binding of its active metabolite 1α,25-dihydroxyvitamin D3, are closely related to retinoid signaling and this modulation is relevant for hematopoiesis and also for AML treatment, as described below. Vitamin D enhances binding of RXR/VDR heterodimer to vitamin D response elements and it restricts the ability of 9-cis-RA to signal through the RXRα receptor, modulating the retinoid signaling pathway [109,110]. 

Vitamin D signaling seems to be dispensable for hematopoiesis, since vitamin D signaling-deficient mice show no phenotype related with blood cells production [111,112,113]. However, this signaling pathway does play a role in modulating this process (reviewed in Bunce et al. [114]) [115], as well as the immune response [112,116,117,118]. Treatment of hematopoietic stem cells or some leukemia cell lines with the active form of the vitamin D leads to increased monocyte/macrophage differentiation [92,119,120], an effect which is not detected in VDR knock-out mice [114,119]. As stated above, when cultured HSC are treated with RA signaling inhibitors, their differentiation is blocked, but retinoids and vitamin D treatment counteract this effect [64]. Since RARα receptor activation induces granulopoiesis, it has been suggested that VDR and RARα can compete for RXR heterodimerization. In this way, VDR/RXR and RAR/RXR heterodimers would drive differentiation of progenitors towards monopoiesis and granulopoiesis, respectively [115,119]. 

ATRA treatment is ineffective for AML, as stated above. Since vitamin D3 induces monocytic differentiation of some lines of leukemic cells, vitamin D analogs could represent a therapeutic option [115,119,121]. Synergy between vitamin D and RA signaling is demonstrated by the enhanced neutrophil differentiation of NB4 cells when a RARα agonist (AGN195183) is used in combination with a low concentration (10 nM) of vitamin D3 [64]. The differentiation effect of the RA/vitamin D3 combination is variable in different AML cell lines [122], and this is probably due to the regulation of the VDR transcription by RARα. High levels of RARα protein repress the VDR gene in absence of RARα agonists [123]. 

## 7. Vitamin A Deficiency and Anemia

Given the role played by vitamin A/RA signaling in hematopoiesis, it seems clear that vitamin A deficiency (VAD) must constitute a serious problem for the functions of the blood system, and this has been demonstrated in a number of animal models. In mice fed with a vitamin A deficient diet or treated with RAR antagonists, an increase of myeloid cells is observed in bone marrow, spleen and peripheral blood [124,125]. This effect leads to a severe splenomegaly in 14-week old mice after having received a vitamin A-deficient diet from birth. This condition can be partially reverted by a vitamin A supplemented diet. The frequency of myeloid progenitors (bone marrow CFUs) is not influenced by the lack of RA, but the frequency of apoptosis in CD11b+/Ly-6G+ neutrophils cells is significantly lower in vitamin A-deprived mice, probably explaining the increased myeloid cell population [124]. On the other hand, rats fed with a vitamin-A deficient diet show a significant decrease in serum iron, transferrin receptor saturation and erythropoietin expression. At the same time, iron concentration increased in the spleen, indicating ineffective erythropoiesis [126]. Furthermore, vitamin A is involved in the regulation of iron-regulatory protein (IRP2), subsequently affecting iron metabolism. In this way, iron deficiency is aggravated in rats under VAD conditions [127]. Interestingly, vitamin A induced ferroportin-1 expression in Caco-2 cells, suggesting that RA signaling could support mobilization and transport of iron from intestinal cells [128].

In humans, particularly in children and pregnant/lactating women, VAD has been recognized as a public health issue in developing countries [129]. The estimated number of people in the world that are affected by micronutrient deficiencies varies between 1 and 2 billion [8,130,131]. Adequate Vitamin A intake is associated with a lower rate of overall morbidity among children [132,133]. VAD (defined as serum retinol <20 µg/dL) is associated with decreased dietary supply of vitamin A and its precursors [131]. An extensive survey of the global extent and importance of VAD is available online [134,135]. Just to quote some facts, an estimated 250 million preschool children suffer from VAD globally. In low-income countries, 10% to 20% of pregnant women develop VAD [136].

Severe VAD leads to xerophthalmia, the most common cause of preventable blindness among children. According to WHO data, 250,000 to 500,000 vitamin A-deficient children become blind every year, half of them dying within 12 months of losing their sight [134]. VAD is also associated with increased risk of death from severe infections [129,137,138]. VAD is also related with anemia, probably through its role in mobilization and transport of iron [139], as suggested by experiments in animal models quoted above [127,128]. Although iron deficiency is the dominant cause of nutritional anemia, simultaneous use of iron and vitamin A supplements seems to be more effective to prevent anemia than the use of either micronutrients alone, a fact that emphasizes the need of studies on the interaction between these micronutrients, especially regarding iron absorption and erythropoiesis modulation [130]. 

The beneficial effects of vitamin A or β-carotene supplements to prevent conditions derived from VAD have been described in a number of papers. Just to mention some instances, biscuits fortified with vitamin A reduced VAD and anemia in Chinese pre-school children [140]. Supplement of vitamin A to women from preconception to lactation in rural Nepal showed that adolescent offspring developed higher levels of circulating antibodies than the control group [141]. This vitamin A treatment also increased circulating hemoglobin during pregnancy, probably by increased insulin-like growth factor-1 (IGF-1), which has functions in erythropoiesis [142]. In Ethiopia, a single high dose of vitamin A (30–60 mg) in children leads to modest increases (1.5 g/L) of circulating hemoglobin six months later, reducing 9% the risk of anemia [143]. Finally, multiple micronutrients in powder (including vitamin A) administered to young Brazilian RA children effectively reduced anemia and improved growth and micronutrient status [144].

A number of strategies can be followed for prevention of VAD, i.e., dietary diversification, high-dose supplementation, conventional food fortification (with artificial additives), biofortification (by conventional plant breeding) and genetic modification of crops [145]. Promotion of dietary diversification through nutrition education programs is a sustainable strategy and it can cover multiple micronutrient deficiencies. However, this strategy lacks of measurable endpoints, and bears issues of affordability [146]. 

Supplementation with high doses of vitamin A periodically dispensed to children between six months and five years of age have demonstrated to be associated with large reductions in mortality, morbidity, and vision problems in an extensive meta-analysis [137]. This conclusion was partially challenged by the DEVTA cluster randomized trial developed in India, where the reduction in mortality by vitamin A supplementation was lower than expected [147]. This result generated a controversy that still continues [137,148,149,150] although periodic vitamin A supplementation seems to be safe and effective considering globally the published reports. 

Fortification of sugar, vegetable oils and cereal-based products by addition of vitamin A has been extensively explored for VAD control in developing countries, having already provided good results in Central and South America [150].

Biofortification of maize by conventional breeding (the “orange” maize) has been developed in Zambia. Orange maize contains enhanced β-carotene concentrations and it appears to be as efficacious as vitamin A supplementation in Zambian children [151,152]. Biofortification of cassava is currently been assayed [153].

Finally, a strategy for fighting against VAD was the introduction of the “golden rice”, a transgenic variety of rice that accumulates β-carotene in its endosperm [154]. Golden rice was created by Ingo Potrykus as a humanitarian project and represents a promising strategy, but the criticisms from a number of environmental and anti-GMO (genetically modified organisms) groups have obstructed their test in humans [155]. 

## 8. Conclusions and Future Directions

In recent years, researchers have become increasingly interested in the multiple functions played by vitamin A/RA signaling in developmental and adult hematopoiesis. This basic knowledge of the molecular mechanisms modulated by RA signaling has led to impressive advances in the therapy of some forms of leukemia, such as the APL. The induction of differentiation of malignant cells has been possible by a better understanding of the cellular and molecular control by RA signaling of myeloid differentiation. A major challenge for the near future will be to overcome the mechanisms of RA resistance in the treatment of other types of leukemia. It will also be very important to advance in the knowledge of the complex interrelationships between vitamin A/RA and vitamin D signaling, particularly in processes related with the modulation of the immune response. Given the critical importance of vitamin A/RA signaling in the immune defense and other essential processes for human health, the fight against vitamin A deficiency is a priority in developing countries. More efforts will be necessary to assess the current practices and to develop new, more effective and efficient strategies to reduce the prevalence of this serious worldwide problem.
